# Pulmonary function testing in COPD: looking beyond the curtain of FEV1

**DOI:** 10.1038/s41533-021-00236-w

**Published:** 2021-05-07

**Authors:** Sotirios Kakavas, Ourania S. Kotsiou, Fotis Perlikos, Maria Mermiri, Georgios Mavrovounis, Konstantinos Gourgoulianis, Ioannis Pantazopoulos

**Affiliations:** 1 Critical Care Department, Sismanogleio General Hospital, Athens, Greece; 2 Department of Respiratory Medicine, University of Thessaly, School of Medicine, University General Hospital of Larisa, Thessaly, Greece; 3 Department of Respiratory Medicine, Evangelismos General Hospital, Athens, Greece; 4 Department of Emergency Medicine, Faculty of Medicine, School of Health Sciences, University of Thessaly, Larisa, Greece

**Keywords:** Chronic obstructive pulmonary disease, Respiratory signs and symptoms

## Abstract

Chronic obstructive pulmonary disease (COPD) management remains challenging due to the high heterogeneity of clinical symptoms and the complex pathophysiological basis of the disease. Airflow limitation, diagnosed by spirometry, remains the cornerstone of the diagnosis. However, the calculation of the forced expiratory volume in the first second (FEV1) alone, has limitations in uncovering the underlying complexity of the disease. Incorporating additional pulmonary function tests (PFTs) in the everyday clinical evaluation of COPD patients, like resting volume, capacity and airway resistance measurements, diffusion capacity measurements, forced oscillation technique, field and cardiopulmonary exercise testing and muscle strength evaluation, may prove essential in tailoring medical management to meet the needs of such a heterogeneous patient population. We aimed to provide a comprehensive overview of the available PFTs, which can be incorporated into the primary care physician’s practice to enhance the efficiency of COPD management.

## Introduction

Chronic obstructive pulmonary disease (COPD) is a progressive inflammatory disease involving the airways, alveoli and pulmonary vasculature, eventually leading to irreversible airflow limitation and loss of elastic recoil. The disease causes a gradual decline in expiratory flow, resulting in increased end-expiratory volume and dynamic hyperinflation^1^. The management of COPD remains challenging, due to the highly heterogeneous nature of the disease, both in its clinical features and its underlying pathophysiological mechanisms^2,3^.The confirmation of chronic airflow limitation using post-bronchodilator spirometry remains a major criterion for the diagnosis of COPD^4^. Furthermore, Forced Expiratory Volume in the first second (FEV1) evaluation is crucial in estimating the severity of airflow limitation and until recently guided the clinical management of the disease. However, it is now widely accepted that FEV1 measurement alone does not effectively represent the functional impairment and subsequent symptomatology experienced by individual patients^5^. A holistic patient assessment including multiple parameters is required for efficient COPD phenotyping, as some phenotypes have been shown to respond to treatment differently and have a worse prognosis. Established phenotypes include frequent exacerbators, patients with predominant emphysema, and patients with overlapping characteristics of asthma and COPD^3^.

The notion of COPD phenotyping and individualized care has been incorporated into the Global Initiative for Chronic Obstructive Lung Disease (GOLD) strategy for the diagnosis and management of the disease. In this context, a broader range of Pulmonary Function Tests (PFTs), comprising of resting lung volume, capacity and airway resistance evaluations, diffusion capacity measurements and field or cardiopulmonary exercise testing provide a more comprehensive assessment of COPD patients. Notably, it has been proposed that the calculation of anthropometric indices could further facilitate the assessment of patients^6^. Nevertheless, the optimal classification of COPD patients remains far from being considered as elucidated.

Each of these parameters highlight different physiological and functional aspects of the disease requiring targeted therapy. The goal of a personalized approach is overcoming the complexity of the disease, in an effort to optimize patient management and eventually improve clinical outcomes. The aim of the current review is to provide a comprehensive overview of the available PFTs, which can be incorporated into the primary care physician’s practice, in order to enhance the efficiency of COPD management.

### PFT phenotyping guides COPD phenotyping differentiation

It has been recognized that COPD is a heterogeneous clinical entity characterized by a variety of pathophysiological processes and functional profiles^7^. The recognition of specific phenotypes in COPD patients is crucial in individualizing and optimizing medical care and may greatly improve their prognosis and quality of life^3^.

In addition to the officially recognized clinical COPD phenotypes, namely chronic bronchitis, predominant emphysemas^3^, frequent exacerbators and the asthma-COPD-overlap (ACO) phenotype, according to GOLD, there are several emerging clinical COPD phenotypes, are expected to be assimilated into daily clinical practice, including the fast decliner^8,9^, the COPD-bronchiectasis overlap phenotype^10^, the systemic phenotype with a high cardiovascular and metabolic comorbidity burden, the pulmonary cachexia phenotype, and the non-smoking COPD phenotype^8^. At this time, PFT patterns alone do not define subsets that respond to particular therapies; however, characteristic features of PFTs contribute to a large extent in COPD phenotyping differentiation.

The predominant emphysema phenotype is a characteristic example of the necessity of COPD phenotyping since patients suffering from emphysema have the lowest survival rate and the highest rate of decline in pulmonary function among COPD patients^11^. The severe airflow limitation is a prominent feature in COPD patients with emphysema/hyperinflation^12^. Patients with upper lobe-predominant emphysema phenotype, dyspnea, and poor exercise tolerance, have been shown to benefit from lung volume reduction surgery (LVRS)^13^. However, the sub-phenotype defined by an FEV1 ≤ 20% of predicted values, and diffusing capacity for carbon monoxide (DLCO) ≤ 20% of predicted values were shown to have lower survival with LVRS^14^. Hence, PFTs can guide optimal disease management.

Another validated phenotype is the group of frequent exacerbators (≥2 per year) who are younger COPD patients without significant cardiovascular morbidities^15^, characterized by extensive airway obstruction and a progressively declining lung function, greater than or equal to 60 ml/year in FEV1, and high mortality^16,17^. The rapid decline in lung function may be identified by the increased residual volume (RV) in patients with a long smoking history^18^. Targeted therapy with daily azithromycin may prove beneficial in this phenotype by decreasing the frequency of exacerbations and, thus, reducing mortality^19^.

Recently, specific guidelines were published concerning the definition, diagnosis and management of ACO. This consensus guidance was developed and endorsed by both GOLD and Global Initiative for Asthma (GINA). Spirometrically, ACO is characterized by moderate, persistent airflow limitation, while post-bronchodilator testing reveals a noticeable but incomplete reversibility^20^. The recognition of this phenotype warrants a different therapeutic strategy as patients with ACO respond better to inhaled corticosteroids and β_2_-agonists compared to those with COPD^21^. The therapeutic significance of this approach is further underlined by the fact that ACO patients suffer from more frequent and severe exacerbations compared with the general COPD population^22^.

A combination of PFTs is required for a specific diagnosis. Among emerging phenotypes^23^, a slightly more severe lung function reduction, along with lower body mass index, respiratory muscle dysfunction, and high hospital admission frequency are noted in patients with COPD and bronchiectasis than those with COPD in isolation^24^. Spirometry alone does not encompass the entire spectrum of pathophysiological changes in this phenotype, given that air trapping and diffusion impairment represent the most common functional abnormalities, not airflow obstruction^25^. The systemic or comorbid COPD phenotype is characterized by milder airflow limitation. However, the greater proportion of cardiometabolic comorbidities are responsible^3^ for more dyspnea, poorer quality of life, increased healthcare utilization and mortality risk than subjects with comparable airflow limitation without a comorbid burden^15^. Exertional dyspnea disproportionate to pulmonary function tests, low carbon monoxide diffusion capacity and rapid decline of arterial oxygenation upon exercise are typical clinical features of the subgroup with poor prognosis^26^. Cachexia in COPD may be prevalent in as many as half of diagnosed patients, especially in the elderly, and is strongly associated with a negative effect on PFTs^27^, significant airflow limitation, symptoms, and exacerbation frequency^3^. Finally, the diagnosis of nonsmoking-COPD, a small-airway disease phenotype with less emphysema, preserved lung diffusion and a slower lung function decline in younger subjects is challenging^28^, given that the assessment of diseased distal small airways requires a combination of several estimates of lung area and volume. Therefore, PFTs can also guide optimal disease modification.

### FEV1

Spirometry is the cornerstone of COPD diagnosis. According to GOLD guidelines, persistent airflow limitation is defined as a post-bronchodilator ratio of FEV1 to Forced Vital Capacity (FEV1/FVC) of less than 0.7^4^. However, the use of a fixed FEV1/FVC ratio as a diagnostic cut-off is a rather simplified approach, leading to overdiagnosis of COPD in older patients, while simultaneously underestimating the presence of COPD in younger patients^29^. Notably, it has been reported that in almost 75% of patients older than 65 years old diagnosed with mild COPD using the GOLD criteria, the measured FEV1/FVC values are within the normal range for healthy age-related and race-matched controls^30^. Thus, although the fixed FEV1/FVC ratio importance has been validated by numerous studies and remains the standard diagnostic criterion due to its simplicity, it should be employed with caution.

### Flow-volume curve concavity

The functional information provided by measuring the FEV1 is limited to the first second of forced exhalation when the small airways are exposed to substantial distending forces. During this period, the small airways’ contribution to total airway resistance is limited, unless extensive airway narrowing is present^31^. In early-stage COPD, when small airway narrowing is not yet extensive, the measurement of FEV1 is not a sensitive marker of airway obstruction^32^.

Spirometry allows visualization of the maximal expiratory flow-volume (MEFV) curve. The concave shape of the MEFV curve has been reported to be useful for diagnosing airway limitation, especially in the poor expiratory effort if the patient cannot exhale for ≥6 sec or create a plateau in the volume-time curve, and it correlates with symptoms^33^. MEFV curves with varying concave contours are seen in patients with COPD and partly attributable to the mechanical interaction between airway conductance and loss of elastic recoil^34^.

The limited small airway narrowing in early-stage COPD is more apparent during the end of exhalation when lung volume is decreased and the distending forces on the small airways are lower^35^. The resulting higher peripheral airway resistance and non-uniform airway emptying produce a characteristic concave pattern at the terminal point of the flow-volume curve, elsewise known as “scooping”^32^. The development of scooping in the descending limb of the flow-volume curve has been recognized as a feature of airflow obstruction, principally concerning the small airways^36–38^. The scooping pattern develops when lung compartments have differing expiratory time constants, causing non-uniform airway emptying, as is the case in early-stage COPD^39^.

It has been suggested that a concave pattern in the flow-volume curve may be the first indication of small airway obstruction in patients with normal FEV1^35^. It has been shown that the visual evaluation of flow-volume curve scooping has been effective in differentiating between healthy adults and patients with mild COPD^40^. Visual evaluation, however, is highly subjective and, thus, several methods of quantifying flow-volume concavity have been proposed^32^. The slope-ratio (SR) index quantifies the slope at desired points along the flow-volume curve^39^ and has been effectively implemented to differentiate between patients with mild COPD and the effects of aging^40^. The quantification of flow-volume concavity has also been attempted by measuring the Forced Expiratory Flow at the point where 50% (FEF_50%_) and 75% (FEF_75%_) of the FVC have been expired, establishing reference values^32,35^. FEF_25%_–_75_% / FVC is another index of dysanapsis to evaluate the curvilinearity of the MEFV curve^41^.

Furthermore, the area under the MEFV curve has been correlated with RV/total lung capacity (TLC), a common index of air trapping^42^ as well as exercise capacity in COPD patients^43^. Moreover, the Obstructive Index, defined as a ratio of FVC to the volume-difference between two points of half of the peak expiratory flow rate (PEFR) on the MEFV curve, has been recently associated with the extent of emphysema, independently of other spirometric indices^44^.

### Peak expiratory and inspiratory flow rate

PEFR reflects the largest expiratory flow rate achieved with a maximally forced effort from a position of maximal inspiration that can be obtained during spirometric recordings. In addition, a peak flow meter can be used in clinical practice to accurately detect COPD in the absence of good quality spirometry^45^. A peak expiratory flow of < 80% predicted is the best cut-off to detect airflow limitation with a 90% sensitivity, and 50% specificity, which further increase in symptomatic patients^45^. Current GOLD recommendations suggest that despite the good sensitivity, PEF measurements alone cannot reliably be used as the only diagnostic test for COPD due to the weak specificity^46^. A positive association between exacerbation frequency and daily PEFR variations has been reported, validating PEFR measurements as a potential monitoring tool in COPD patients^47^. Airway inflammation has also been associated with PEFR variability^48^.

On the other hand, the peak inspiratory flow rate (PIFR), which is the maximum flow rate obtained during an inspiratory maneuver, is regularly measured without resistance during standard pulmonary function testing. More specifically, spirometry contributes to the identification of patients with a decreased maximal inspiratory pressure denoted FIFmax based on gender and height during a forced inspiratory vital capacity maneuver^49^. FIF max has been shown to correlate with PIFR. Alternatively, PIFR can be assessed by inspiratory devices that enable healthcare professionals to coach patients to use their inhalers correctly.

Inhaled drug delivery is the cornerstone for COPD treatment, preventing exacerbations and hospitalization^50^. The optimal drug delivery to distal airways and lung parenchyma is associated with PIFR. The optimal PIFR for adequate drug dispersal from a DPI is reported to be greater than 60 L/min for low resistance devices^51^, and the minimal inspiratory flow rate is reported to be 30 L/min for high resistance inhalers.

There is no clear correlation between PIFR and FEV1^52^. PIFR can be reduced at the time of COPD exacerbation. COPD patients with suboptimal PIFR were found to have increased levels of dyspnea complaints, exercise limitation and less mean time to the next exacerbation than patients with optimal PIFR^53^. The strength and endurance of inspiratory muscles is one determinant of PIFR^54,55^. Low inspiratory muscle strength is associated with suboptimal PIFR. Recent research suggested a relationship between skeletal muscle strength of the hand and PIFR in hospitalized patients with acute exacerbations. Furthermore, insufficient PIFR measurements were more prevalent in females, patients with air trapping or short stature among ambulatory patients^56^.

### No spirometric measure correlated with arterial blood gases

It is accepted that no spirometric parameter correlated with resting arterial blood gases. Spirometry involves dynamic measurements of the bellows function of the lung. On the other hand, arterial blood gas status is dictated by several factors, including ventilation/perfusion (V̇/Q) ratio, and should be determined invasively by measurement in each individual patient^56,57^. Arterial blood gas analysis is indicated for COPD patients, independently of COPD severity, at rest or acute exacerbations to detect hypoxemia and/or hypercapnic acidosis, which are strong determinants of COPD survival.

### Resting lung volumes and capacities

Multiple techniques are available for the measurement of lung volumes. No one method should be promoted over the others. Nevertheless, whole-body plethysmography (WBP) is often considered more accurate than gas dilution methods in the presence of obstruction. WBP has been recognized as a technique that overcomes problems of maldistribution of gas within the lung and yields a higher FRC in patients with obstructive lung disease as plethysmographic FRC includes well-ventilated as well as poorly ventilated areas of the lung. Contradictory data are also present, suggesting that WBP-derived TLC could overestimate the lung volume, especially in cases of severe obstruction^58^. On the other hand, lung volumes measured by nitrogen washout or He dilution are typically smaller than lung volumes measured by WBP when air trapping is present^59^, as it is difficult for the gas to reach all the lung areas.

**Fig. 1:**
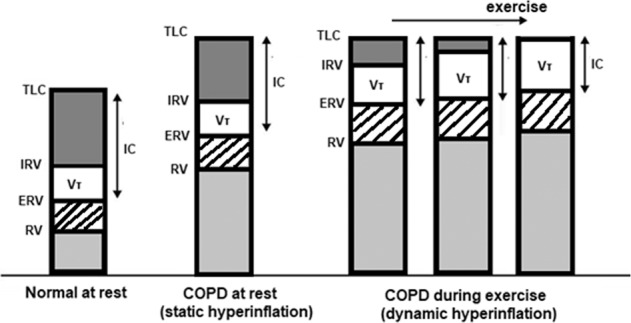
Pulmonary Volumes during rest and exercise in COPD patients. Figure depicting pulmonary volumes in COPD patients during rest and exercise, representing static and dynamic inflation respectively.

The key pathophysiological feature of COPD is undoubtedly expiratory airflow limitation, which may result in air trapping and lung hyperinflation, manifested as increased operating lung volumes and reduced Inspiratory Capacity (IC) and Inspiratory Reserve Volume (IRV)^60^ (Fig. 1). WBP uses Boyle’s law to measure the intrathoracic gas volume or functional residual capacity (FRC), and once this is determined, the RV and TLC are extrapolated. TLC, FRC, and RV are considered normal when they range between 80% and 120% of the predicted value. Static lung volumes have been associated with the sensation of dyspnea and the higher metabolic demands of ventilation in patients with COPD^60^.

RV is generally the first volume to increase in obstructive lung disease and can be a good measure to evaluate early disease states^61^. In the case of forced expiration, air trapping is reflected by an increase in the RV within a slightly preserved TLC. The RV/TLC ratio, which reflects the proportion of trapped lung volume that cannot be mobilized by maximal breathing represent, is the most reliable parameter in assessing air trapping and hyperinflation^62^, together with increases of RV and FRC. On the other hand, TLC, RV, and RV/TLC are significantly increased as COPD is exacerbated and airflow limitation worsens^63^. Previous studies have shown that an increase of RV/TLC was a powerful predictor of all-cause mortality and frequent exacerbations in the COPD population^64^. A RV/TLC ratio greater than 60% is used as a criterion for severe hyperinflation and a prerequisite for referral to LVRS^65^. Lung volume is also very sensitive to bronchodilators and lung-volume reduction surgery and better associated with patient-centered outcomes such as dyspnea and exercise tolerance. Preoperative RV/TLC ratio was reported to be predictive of postoperative outcomes after lung-volume reduction surgery^65^.

Another indicator of severe hyperinflation is a severe reduction of resting IC or IC/TLC when TLC is stable^60^. Resting IC is strongly associated with static lung hyperinflation and the elastic load that inspiratory muscles receive. The decrease of IC linearly correlates with the degree of airflow limitation^66^. Thus, IC is a sensitive marker of the severity of COPD and may indicate flow limitation even in patients with milder disease, in whom FEV1 is preserved. From a functional point of view, resting IC may predict patients’ tolerance to exercise. IC/TLC < 25% is a predictor of mortality, exercise impairment, and exacerbation in COPD patients^67^.

In hyperinflated COPD patients, the measurement of static lung volumes and capacities reveals a lower inspiratory fraction, measured as the ratio of IC to Total Lung Capacity (IC/TLC)^68^. Inspiratory fraction is a predictor of mortality and respiratory failure^69^. The devastating consequences of hyperinflation eventually lead to worsening neuromechanical dissociation and dyspnea^70^. Therefore, it is of great clinical importance to measure static volumes and capacities in patients with COPD in order to guide and evaluate the effectiveness of therapeutic interventions. The use of bronchodilators can potentially increase IC, reduce dyspnea ratings and increase exercise tolerance^71^.

### Airway resistance and specific airway resistance

WBP also allows the assessment of airway resistance (Raw) during tidal breathing in a closed body box^72^. Raw is a measure of airway obstruction, defined as the relationship between airflow in the respiratory tract and the changes in the alveolar pressure required to establish a flow rate of 1 L s^−721^. The airflow needs a generation of pressure. The greater the pressure is required for a given flow, the greater the resistance. More specifically, Raw is defined as the ratio of driving pressure (the difference of alveolar and mouth pressure which is essentially constant during unimpeded breathing) and the flow rate determined at the mouth or the ratio of sRaw to FRC.

It is important to realize that the primary measure recorded in WBP is specific Raw (sRaw) which is, despite its name, not a resistance. sRaw is described as the work to be done by volume displacement (shift volume) to establish a flow rate. The ratio of shift volume to flow rate, representing sRaw.

There is an increase in sRaw and Raw accompanied by a reduced tidal flow in obstructive lung disease^73^. SRaw has been linked to activity-related dyspnea in moderate to severe COPD patients^72^. The relative decrease of sRaw following bronchodilator inhalation has been reported to cover changes in both lung hyperinflation and airway resistance, which might be underestimated by spirometry.

If the airflow is plotted on the vertical axis versus shift volume on the horizontal axis, closed curves are obtained. The reciprocal slope of these breathing loops represents sRaw. In healthy subjects, the curves are approximately straight lines, while in patients with obstructive respiratory diseases, altered patterns can be recognized that carry information on the particular disorder and are helpful in the differential diagnosis.

Analysis of the WBP breathing loops contains potentially relevant information about the pathophysiology of COPD. Any flattening indicates obstruction, which might be different for inspiration and expiration. COPD patients exhibited substantially enlarged loops^25^ when tested during tidal breathing compared to healthy subjects^74^. Recently, it has been reported that in the majority of COPD subjects with hyperinflation, loops are mathematically identified by an open appearance as measured by roundness that typically occurs. In contrast, the rotation of the slope without opening is apparent during bronchoconstriction, as demonstrated in asthma^74^. Opening of the loops in COPD indicating trapped air, has been significantly associated with RV/TLC ratio.

Mechanistic and prospective follow-up studies are needed for a more detailed evaluation of the opening of loops to reveal valuable information in COPD and determine the true validity of these parameters.

It should be noted that although WBP is particularly advisable when patients are unable to perform the spirometry maneuvers as needed and is clinically useful for indicating severe hyperinflation, access to WBP is unavailable in many clinical situations, such as primary care.

### Diffusion capacity

Gas exchange through the functional lung surface can be globally assessed by DLCO, measured by various methods. In everyday clinical practice, the single breath method has been established as the method of choice, providing well standardized predictive norms^75^.

In principle, a low DLCO is indicative of the emphysematous phenotype of COPD^14^. In these patients, rapid lung function decline is independently associated with the severity of emphysema, as assessed by either Computed Tomography (CT) or DLCO^17^. Furthermore, a preserved DLCO is a reliable predictor of exertional desaturation in COPD patients. DLCO has a clear relationship with functional and clinical outcomes in COPD, and its impairment has been recently described as a good predictor of 6MWT decline and was closely correlated with the mean walking intensity^69^. Considering that neither abnormal resting oxygen saturation nor pathologic pulmonary function studies are indicative of exercise-induced oxygen desaturation, the information provided by DLCO can be valuable in the clinical assessment of patients with emphysema^76^.

COPD is usually associated with mild pulmonary hypertension. However, a subgroup of patients develops severe precapillary pulmonary hypertension disproportionate to the degree of underlying airflow limitation^77^. These patients suffer from severe, progressive dyspnea and chest CT reveals diffuse emphysema^78^. Spirometry is normal or may show mild limitation, but DLCO is severely reduced^79^.The decrease in DLCO may result from a fall in alveolar volume (VA), representing the volume of CO distribution during the breath-holding maneuver mainly due to restrictive and/or obstructive defects and/or a fall in carbon monoxide transfer coefficient (KCO). While DLCO is a measure of total lung diffusing capacity, the VA, when corrected for TLC (VA/TLC), represents the fraction of accessible inspiratory lung volume and provides valuable information about inspired gas distribution abnormalities and, therefore ventilation inhomogeneity. Hence, adjusting predicted DLCO for alveolar volume provides a better assessment of lung function^80^. KCO or DLCO/VA is considered an index to assess the efficiency of alveolar transfer of carbon monoxide by measuring the pulmonary gas exchange across the alveolar–capillary membrane during breath-holding at full inflation in a single-breath measurement^81,82^. KCO reflects changes in functional lung volume and impairment in gas transport across the alveolar–capillary membrane. Its increase is related to parenchymal destruction, small airway disease and microvascular destruction^81^.

KCO so far has earned limited clinical consideration in routine pulmonary function testing in COPD. However, emerging data supported^83^ that the decline in KCO better reflects emphysematous changes in COPD compared to DLCO^84^, and it should be considered as an important outcome measure in future clinical studies. More specifically, one of the most recent studies found that the decline of KCO was associated with disease severity in terms of airflow limitation and emphysema, as well as with an accelerated decline in FEV1, more frequent exacerbations, thus reflecting worse disease outcomes^85^. Besides, a decreased KCO has been associated with increased pulmonary venous pressure and cardiac problems that affect the prognosis of COPD. It should be noted that FEV1 alone does not adequately predict disease prognosis.

Similar to DLCO, nitric oxide (NO) diffusing capacity (DLNO) obey Fick’s First Law of Diffusion and the basic principles of chemical kinetic theory. NO gas transfer is dominated by membrane diffusion, whereas CO transfer is limited by diffusion plus chemical reaction within the red cell. Hence, NO has a much stronger binding affinity for hemoglobin than CO. Therefore, the DLNO is independent of pulmonary capillary blood volume and equals the true membrane diffusing capacity^86^. It has been supported that the DLNO could be more sensitive in detecting alveolar destruction than the DLCO. A previous study supported that KNO (DLNO/VA) has a slightly higher sensitivity to detect emphysema than the KCO and FEV1/FVC in heavy smokers, with a high negative predictive value 98.2%, indicating an emphysema exclusion test^86^. Although DLNO has been the topic of research over the last 15 years, applying the DLNO-CO technique in daily settings is still limited by some technical drawbacks^87^.

### Oscillometry

Commercialized forced oscillation technique (FOT) devices to assess impedance in obstructive diseases such as COPD have gained popularity. Spirometry requires forced maximal maneuvers but cannot quantify increased airways’ resistance at tidal breathing, which may be characteristic and specific for COPD. On the contrary, FOT did not require forced expiratory maneuvers and can be utilized to evaluate lung function in patients unable to perform spirometry, including children and elderly patients^88^. The FOT is a noninvasive method of estimating lung mechanics by delivering sinusoidal waves in the opening of the airway through a mouthpiece during spontaneous ventilation^89^.

FOT application provides detailed information about the lungs’ resistive properties, reflecting changes in lung structure, heterogeneity and elastance^44^. FOT is used to estimate the respiratory system impedance (Zrs), which includes the respiratory system resistance (Rrs) and respiratory system reactance (Xrs)^42,43^. Resistance can be conceptualized as a sound wave that requires to travel through the airways and inflate the lung^90^. The reactance of the respiratory system is composed of the inert and elastic properties of the respiratory system. The sum of the forces ahead of the sound wave (resistance) and those generated behind the sound wave in response to the pressure of the wave (reactance) equal the impedance of the entire respiratory system, thus, permits passive measurement of lung mechanics^90^.

The earlier FOT devices allowed only one sound frequency to be passed at a time. The more recent FOTs now use sound waves of two or three different frequencies at one time^91^. Impulse oscillometer (IOS) is an improvised technique of that FOT that could use multiple sound frequencies at one time^92^. Although FOT devices are generally comparable, IOS is the most commonly used type of FOT in clinical practice^93^. IOS is a convenient technique for evaluating lung function and peripheral airway dysfunction during tidal breathing by an effort-independent and patient-friendly modality^94^, providing an extensive description of oscillatory pressure and giving better mathematical analyses of resistance and reactance using the fast Fourier transform (FFT) technique compared to FOT. In IOS, an impulse, which can be mathematically decomposed into different frequencies, is transmitted, whereas, in FOTs, the sound waves of different frequencies were transmitted sequentially. This reduces the test duration and provides a high signal to noise resolution^94^.

Small airway disease is a silent signature of early COPD that is likely to be directly or indirectly captured by combinations of physiological tests, such as spirometry, WBP, IOS, and multiple breath nitrogen washout. However, for the clinical practice, it is important to adopt easy-to-use clinical measures, such as IOS, to delineate early COPD. Oscillometry is more sensitive than spirometry in identifying peripheral airway pathology^43,45^. Importantly, IOS can differentiate small airway obstruction from large airway obstruction and is more sensitive than spirometry for peripheral airway disease^90^.

Notably, it can detect early lung dysfunction in smokers, long before any clinical symptoms of COPD arise^95^. FOT findings in COPD consist of an increased Rrs and a decreased Xrs value, culminating in an increased expiratory Zrs^89^. These findings are suggestive of small airway obstruction and, importantly, Zrs values correlate to the severity of airway narrowing^96^. Furthermore, FOT is more sensitive than FEV1 in detecting expiratory flow limitation, an indicator of dynamic hyperinflation characteristically present in COPD patients during exertion^97^. It has been recently suggested that IOS may be used alternatively to other PFTs in patients with FEV1 lower than 50%^98^. Yamagami *et al*., proved that FOT parameters differ between COPD patients with frequent exacerbations and those having a less eventful disease course. Consequently, patients identified as frequent exacerbators can be monitored more closely and treated accordingly^99^. In the Eclipse study, one of the largest COPD studies, with a 2,054 cohort of COPD patients, it has been shown that changes in oscillometric parameters tend to correlate well with GOLD severity.

Lately, oscillometry became a promising tool in lung mechanics’ assessment. However, there are substantial limitations of this method. Reference values for COPD were studied, but there are no defined values yet; hence, they are often difficult to interpret. Standardization documents still need improvement, especially on normative values. Also, the comparison between different devices requires further study. In the absence of this data, the ERS Task Force recommends that reference values should be derived from the most similar to the device in use^100^. These limitations will probably prevent their acceptance by physicians. The development of new easier to use and portable devices and the acquired knowledge on interpreting oscillometry parameters will hopefully make this technique more accepted and useful for daily practice. Whether forced oscillation techniques will find their way into clinical practice in which WBP is available, remains to be shown.

### Field exercise tests

The evaluation of the functional status of COPD patients is of great clinical importance, as it guides the selection of medical therapy and pulmonary rehabilitation programs^101^. The 6 min walking test (6MWT) is a submaximal exercise test that can be easily performed and is well tolerated by patients. 6MWT has been reported to be a good indicator of functional capacity in relation to exercise in COPD patients^102^. Studies have also shown a good correlation of 6MWT to peak oxygen uptake^103^. Moreover, it is considered to be more representative of the patients’ daily activity, when compared to other field or cardiopulmonary exercise tests^104^. A reduced 6MWT distance is an adequate index of functional disability and an increased mortality risk, although no predictions can be made regarding hospitalizations due to COPD exacerbations^103,105^. As a result, 6MWT has been tested and included in the Body mass index, Obstruction, Dyspnea, and Exercise capacity (BODE) index^106^. This compound prognostic index predicts mortality risk in COPD patients and was initially developed in a large cohort of patients at various stages of COPD^106^. In this cohort, patients who died within 1 year achieved significantly shorter 6MWT distances compared with survivors (mean ± standard deviation 175 ± 86 m vs 264 ± 113 m; P = 0.001). BODE index is also significantly higher in frequent exacerbators compared with infrequent exacerbators^9^.

Various other field exercise tests have been validated in the COPD population as alternatives to 6MWT. Similarly to the 6MWT, the shuttle walking test was developed to simulate a cardiopulmonary exercise test using a field walking procedure. This test is used to assess the exercise capacity of COPD patients, while it has been reported as being more responsive to the effect of pulmonary rehabilitation training^107^.

The Sit-to-Stand Test can be used to determine the functional capacity in COPD patients, especially those who are older or markedly disabled^108^. In these patients, standing up from a sitting position represents an essential activity, which is vital for maintaining independence^109,110^.

### Cardiopulmonary exercise testing (CPET)

Cardiopulmonary exercise testing (CPET) is a more sophisticated method of assessing functional capacity and impairment in COPD patients^111^. In contrast to volitional exercise field tests, CPET requires advanced equipment as well as expertise and experience and evaluates both the submaximal and peak exercise responses. CPET provides an integrated assessment of the exercise response involving the pulmonary, cardiovascular, neurosensory, and musculoskeletal systems^111^. Basic ventilatory measurements include gas exchange in terms of respiratory oxygen uptake (VO2) and carbon dioxide production (VCO2), minute ventilation and other dynamic ventilatory measurements, during a symptom‐limited exercise test. When these parameters are combined with metabolic and cardiovascular indices, a series of other variables can be derived^112^.

Exertional dyspnea is the most common symptom in COPD patients, signaling reduced exercise capacity and leading to physical inactivity, which has been associated with reduced survival^113^. Thus, the amelioration of dyspnea and exercise tolerance are uniformly recommended by expert guideline committees^114^. Importantly, it has been clearly established that patients can suffer from exertional dyspnea independently of the severity of airway obstruction^115^. In the everyday clinical practice, the magnitude of patients’ respiratory discomfort when confronted with physical tasks is evaluated by the use of simple questionnaires. However, these simplified assessments can lead to a substantial underestimation of the actual degree of exertional dyspnea in some patients. For example, it has been shown that a large number of COPD patients exhibit marked respiratory discomfort during CPET at low-work intensities, although they initially deny suffering from significant activity-related dyspnea^116^. Furthermore, traditional resting PFTs have failed to provide indices that directly correlate with the severity of exertional dyspnea^117^.

Previous studies have delineated a COPD phenotype characterized by mild airway obstruction but disproportionately severe dyspnea^115^. This subgroup of patients is adequately detected by the use of CPET^117^. CPET can also reveal dynamic physiological abnormalities in smokers with normal spirometry who present with activity related respiratory discomfort^118^. In the general COPD population, peak oxygen uptake measured by CPET correlates with survival^119^.

During CPET protocols, the resting physiological abnormalities of COPD patients become amplified. Intrinsic positive end-expiratory pressure (PEEP) leads to increased elastic loading and decreased dynamic lung compliance, while the resistive loading of the respiratory muscles increases^111^. As a result, CPET-integrated monitoring detects an increased efferent respiratory drive, an intense stimulation of central and peripheral chemoreceptors and an increased contractile respiratory muscle effort. IC and IRV decrease, and as TLC is approached, further tidal volume expansion is limited (Fig. 1). The generation of resting tidal volume requires an unequally intense respiratory effort, thus leading to respiratory discomfort. In the advanced stages of COPD, these phenomena become evident at a progressively lower exercise intensity and the exercise is terminated when the minimal possible IRV is reached, because a further increase in ventilation is impossible. Due to dynamic lung hyperinflation, the respiratory muscles fail to respond adequately to the continuously increasing central drive to breathe.

Lung hyperinflation and diminished resting IC in patients with COPD can be ameliorated by the use of both classes of inhaled bronchodilators, namely β2-agonists and muscarinic antagonists^120,121^. It has been shown that these agents increase resting IC by approximately 0.2–0.4 L or 10–15%^121,122^. Consequently, the available IRV is also increased and, thus, bronchodilators facilitate the necessary tidal volume expansion during exercise^120,123^. The expansion of tidal volume is achieved by less central neural drive and respiratory muscle effort. Thus, neuromechanical dissociation which leads to intolerable dyspnea is delayed and exercise capacity increases^123^. Noteworthily, previous studies have reported that bronchodilator therapy increases the cycle exercise endurance time by 20% in COPD patients^120,121,124,125^.

The administration of supplemental oxygen has been shown to improve dyspnea and exercise tolerance by decreasing central respiratory drive in selected COPD patients^126^. This intervention is associated with reduced respiratory rate (and increased expiratory time) and usually delayed dynamic hyperinflation^127,128^. Supplemental oxygen may also delay ventilatory stimulation due to metabolic acidosis, by facilitating oxygen delivery and utilization at peripheral tissues^129,130^. Similar beneficial effects have been reported with opioid therapy in selected COPD patients^131,132^. However, according to a meta-analysis on the efficacy of low-dose opiates in COPD patients, exercise capacity was not improved, even though dyspnea was reduced^133^. Anxiolytic agents may be considered in predominantly anxious patients in an attempt to ameliorate dyspnea during pulmonary rehabilitation^134^. In this way, the increased central neural drive and the related neuromechanical dissociation may be delayed, slowing down the onset of intolerable dyspnea^135^.

### Anthropometric measures

In patients with COPD, malnutrition and sarcopenia are often present and correlate with the severity of airway obstruction^136^. The final result, previously termed “pulmonary cachexia syndrome”, is associated with sedentary lifestyle and an accelerated decline in functional status^137^. Therefore, patients should be screened for the presence of pulmonary malnutrition during routine visits. Malnutrition can be diagnosed based on the presence of a body mass index (BMI) of less than 18.5 kg/m^2^ or based on weight loss (more than 10% irrespective of time or more than 5% over the last 3 months) combined with a reduced BMI (less than 20 kg/m^2^ or less than 22 kg/m^2^ if 70 years old or older) or a reduced gender-dependent Fat Free Mass Index (FFMI less than 15 or 17 kg/m^2^ in women and men, respectively)^138^.

Sarcopenia forms a part of the cachexia syndrome. Its diagnosis requires the presence of low muscle mass (Appendicular Lean Mass of less than 7.23 for men and less than 5.67 kg/m^2^ for women) in combination with low muscle strength (handgrip strength of less than 30 kg for men or less than 20 kg for women) or low physical performance (gait speed less than 0.8 m/s)^138^. It is important to emphasize that it can occur without concomitant weight loss if fat mass is preserved as the proportion of lean body mass decreases. Reduced muscle mass indices are indicative of the combined effects of muscle deconditioning, poor nutrition and inflammation, associated with the progression of COPD and its comorbidities^139^. Thus, it is not surprising that a decreased FFMI is a stronger predictor of COPD-related physical disability and mortality than low BMI alone^140^.

The cachexia mechanisms underlie muscle^137^ impairment of (inspiratory and expiratory) respiratory muscles in COPD. However, the assessment of respiratory muscle strength is not widely known. There are several methods used to assess respiratory muscle strength during the inspiratory and expiratory phases of respiration, divided into volitional tests that rely on patient understanding and cooperation; such as those that measure maximal inspiratory and expiratory pressures, and non-volitional tests depend on magnetic phrenic stimulation accompanied by the measurement of inspiratory mouth pressure, inspiratory transdiaphragmatic pressure, or inspiratory esophageal pressure. Another emerging noninvasive method introduced is ultrasound imaging of the diaphragm. Volitional tests that measure maximal inspiratory and expiratory pressures are the most commonly used in COPD patients because they are readily available. We believe that primary care physicians involved in the care of patients with respiratory diseases should be familiar with the tests used to assess respiratory muscle function.

### Most commonly used measures to assess respiratory muscle strength

Maximal inspiratory pressure (MIP) is measured via a low-cost, portable analog or digital pressure manometer and by using an easy maneuver, which depends on patient cooperation. Patients are in a sitting position, with or without nose clips, and asked to exhale to RV and then perform a maximal inspiratory effort, sustaining it for 1 to 2 s. MIP is important for diagnosing inspiratory muscle weakness in COPD, the differential diagnosis of obstructive lung disease of unclear origin; assessing response to cardiopulmonary physiotherapy and rehabilitation; prescribing and monitoring respiratory muscle training^141^. The reference values are well-established in different populations (lower limit of normal of 60 cmH_2_O for females and 80 cmH_2_O for males). Previously it has been suggested that MIP was positively correlated with the distance walked in 6MWT in COPD patients^142^. However, according to Kofod et al, whilst MIP was associated with leg muscle strength, it was not associated with walking distance or symptoms^143^.

The sustained maximal inspiratory pressure (SMIP) provides a more robust assessment of inspiratory muscle function as it reflects single breathwork capacity by capturing the amount of pressure generated during a sustained maximal inspiratory effort performed following a full expiration, from RV to TLC^141^. SMIP has recently been validated for use in COPD and has been associated with functional exercise capacity and dyspnea, more severe airflow obstruction, greater hyperinflation, worse health and mental status and impaired quality of life in COPD individuals^144^.

Sniff nasal inspiratory pressure (SNIP) measures the diaphragm activity and selective contraction of the inspiratory accessory muscles^145^, demonstrating the test’s specificity. It also accurately reflects esophageal pressure (Pes), having the advantage of being noninvasive and reproducible^146^. It uses pressure manometers that also measures MIP and has well-established reference values in different populations (lower limit of normal of 70 cmH_2_O for males and 60 cmH_2_O for females^147^; The maneuver is usually performed in sitting position, but it can be performed in any body position^147^. The one nostril should be absolutely patent, whereas the other nostril should be completely closed by a nose plug to prevent pressure from leaking. The maneuver begins with a fast deep inspiration from FRC and the mouth closed after a period of quiet breathing. The use of both MIP and SNIP reduces the rate of false-positive results for respiratory muscle weakness by nearly 20%^148^. SNIP is significantly related to COPD severity as assessed by the BODE index^149^. It has also been reported that the SNIP conveys at least as much predictive power for mortality in COPD as hyperinflation determined by IC/TLC ratio being cheaper, quicker and easier than measuring lung volumes by plethysmography^150^.

Inspiratory mouth pressure (Pm) is recorded by a pressure sensor attached to a mouthpiece (as in the MIP and SNIP), but it does not differentiate between which respiratory muscle is affected. It is mostly used as an indirect measure of esophageal pressure (Pes) during a sniff to confirm inspiratory muscle weakness in COPD patients. The PaCO2 was found to be inversely correlated with Pmax^151^.

Maximal expiratory pressure (MEP) is the most commonly used method for measuring expiratory muscle strength in critically ill patients and outpatients^152^. Patients perform a maximal expiratory effort, sustaining it for 1 to 2 s. It is useful in assessing cough strength, given that one of the phases of cough is the explosive expiration. Expiratory muscle weakness correlates with respiratory infections^153^. Recently, it has been reported that in moderate to very severe COPD patients the sarcopenia could be predicted by FEV1 (%predicted) < 52, MIP < 73 cmH_2_O, MEP < 126 cmH_2_O and distance traveled of <295 m in incremental shuttle walk test. Of note, expiratory and inspiratory muscle training devices significantly improved respiratory muscle function in patients with mild to very severe COPD^154^.

An additional test to assess expiratory muscle weakness is measuring cough gastric pressure (Pga), because the abdominal muscles are mainly responsible for expiratory flow during a cough^141^. It has well-established reference values, greater specificity and negative predictive value than MEP. Its main disadvantage is that it is an invasive method that requires the insertion of a catheter with a pressure sensor into the stomach^141^.

Regular exercise is a natural anabolic modality that not only inhibits muscle atrophy, but also ameliorates dyspnea in patients with COPD^155^. Nutritional supplementation has been shown to increase body weight and fat-free mass in malnourished patients with COPD, resulting in improved peripheral and respiratory muscle strength, exercise tolerance and overall health-related quality of life^156^. It should be noted that well-nourished patients can also benefit from nutritional support as part of an exercise rehabilitation program^157^.

A summary of the available PFTs for the evaluation of COPD patients is depicted in Table 1.

**Table 1:** Pulmonary function tests for the evaluation of COPD patients.

Pulmonary function test	Indicator of airflow limitation	Advantages
FEV1/FVC	Decreased FEV1/FVC Decreased FEV1	Gold-standard Reflects severity of airflow limitation.
Flow-volume curve	Scooping	First indication of small airway obstruction in patients with normal FEV1. Effective in differentiating between healthy adults and patients with mild COPD.
PEFR	A PEFR of < 80% predicted detects airflow limitation (90% sensitivity, 50% specificity)	PEFR measurements alone cannot reliably be used as the only diagnostic test for COPD due to the weak specificity. A potential monitoring tool
PIFR	No clear correlation between airflow limitation or FEV1 and PIFR	PIFR is related to optimal drug delivery with dry powder inhalers used to treat COPD. It can be reduced at the time of COPD exacerbation.
Resting lung volumes and capacities	Decreased IC	May indicate flow limitation even in patients with milder disease, in whom FEV1 is preserved. Resting IC may predict patients’ tolerance to exercise. IC/TLC is a predictor of mortality and respiratory failure.
Specific airway resistance (Sraw) Breathing loops	Increased specific airway resistance Flattening and open appearance	Opening in breathing loops indicate airway obstruction, trapped air, and it has been significantly associated with RV/TLC ratio.
DLCO	Low DLCO	Indicative of emphysema. Predictor of exertional hypoxemia.
KCO	Low KCO	KCO better reflects emphysema compared to DLCO. Predictor of COPD exacerbations and outcome
DLNO	Low DLNO	DLNO could be more sensitive in detecting alveolar destruction and emphysema than the DLCO or KCO.
Oscillometry	Increased expiratory Zrs	Can detect lung dysfunction in smokers, before any symptoms arise and small airway disease Zrs correlates to the severity of airway narrowing. More sensitive than FEV1 in detecting expiratory flow limitation.
Field exercise tests (6 MW test)	Decreased distance achieved	Representative of the day-to-day physical activity of the patients. Correlates with mortality.
CPET	Combines various parameters measured during a symptom-limited exercise test (e.g., decreased VO_2,_ decreased SpO_2_, decreased TV)	Adequately detects patients with mild airway obstruction but disproportionately severe dyspnea. Can reveal dynamic physiological abnormalities in smokers with normal spirometry.
Anthropometry	Malnutrition Sarcopenia (low muscle mass)	Correlate with the severity of airway obstruction. Indicative of higher mortality.
MIP	Low MIP	Indicative of inspiratory muscle weakness
SMIF	Low SMIF	Associated with functional exercise capacity, dyspnea, airflow obstruction, greater hyperinflation, worse health and mental status and impaired quality of life
SNIP	Low SNIP	Impaired diaphragm activity and inspiratory accessory muscle dysgunction Related to severity Predictor of Mortality
MEP	Low MEP	Expiratory muscle weakness

Table summarizing the available pulmonary function tests for the evaluation and classification of COPD patients.

*COPD* Chronic Obstructive Pulmonary Disease, *CPET* Cardiopulmonary exercise testing, *DLNO* nitric oxide diffusing capacity, *FEV1* Forced. Expiratory Volume in the first second, *FVC* Forced Vital Capacity, *IC* Inspiratory Capacity, *KCO* carbon monoxide transfer coefficient, *MEP* Maximal expiratory pressure, *MIP* Maximum inspiratory pressure, *TLC* Total Lung Capacity, *DLCO* Lung Diffusing Capacity for carbon monoxide, *Zrs* respiratory system impedance, *6MWT* 6 min walking test, *PEFR* Peak expiratory flow rate, *PIFR* Peak inspiratory flow rate, *SMIP* Sustained maximal inspiratory pressure, *SNIP* Sniff nasal inspiratory pressure, *VO2* respiratory oxygen uptake.

## Conclusion

Undoubtedly, FEV1 remains an invaluable tool for COPD diagnosis and management. However additional functional testing is necessary in order to tailor COPD management to the variety of symptoms and phenotypes encountered in the everyday clinical practice. The armamentarium of the up-to-date pulmonologist should include resting volume and capacity measurements, lung diffusion testing measures, oscillometry, field and cardiopulmonary exercise testing and muscle strength evaluation. Finally, a personalized approach, guided by the results of PFTs, should be followed in order to optimize patient care.

## Methods

A computerized database search was performed. We searched for published and articles ahead of print in bibliographic databases, including ISI Web of Science, PubMed, Science Direct Scopus, Wiley online library, Google Scholar and other international databases and websites. The literature search also involved a manual review of the bibliography of the identified papers and relevant information to ensure the objectives of this study were met. Keywords used in the search were: “COPD” AND “spirometry”, “pulmonary function tests”, “lung volumes”, “lung capacities”, “diffusion capacity”, “forced oscillation technique”, “cardiopulmonary exercise testing”, “6 min walking test” “exercise tests” “anthropometric indices”. The literature was limited to journal articles written in English. The last update of the search was performed in January 2021.

### Reporting summary

Further information on research design is available in the Nature Research Reporting Summary linked to this article.

## Supplementary information

Reporting Summary

## Data Availability

No original data were included in our article.

## Code availability

None was utilized.
